# Imaging methods to evaluate tumor microenvironment factors affecting nanoparticle drug delivery and antitumor response

**DOI:** 10.20517/cdr.2020.94

**Published:** 2021-06-19

**Authors:** Amber S. Moody, Paul A. Dayton, William C. Zamboni

**Affiliations:** 1 UNC Eshelman School of Pharmacy, University of North Carolina, Chapel Hill, NC 27599, USA.; 2 UNC Lineberger Comprehensive Cancer Center, Chapel Hill, NC 27599, USA.; 3 Carolina Institute for Nanomedicine, Chapel Hill, NC 27599, USA.; 4 Joint Department of Biomedical Engineering, University of North Carolina and North Carolina State University, Chapel Hill, NC 27599, USA.

**Keywords:** Tumor microenvironment, imaging, drug delivery, nanoparticles

## Abstract

Standard small molecule and nanoparticulate chemotherapies are used for cancer treatment; however, their effectiveness remains highly variable. One reason for this variable response is hypothesized to be due to nonspecific drug distribution and heterogeneity of the tumor microenvironment, which affect tumor delivery of the agents. Nanoparticle drugs have many theoretical advantages, but due to variability in tumor microenvironment (TME) factors, the overall drug delivery to tumors and associated antitumor response are low. The nanotechnology field would greatly benefit from a thorough analysis of the TME factors that create these physiological barriers to tumor delivery and treatment in preclinical models and in patients. Thus, there is a need to develop methods that can be used to reveal the content of the TME, determine how these TME factors affect drug delivery, and modulate TME factors to increase the tumor delivery and efficacy of nanoparticles. In this review, we will discuss TME factors involved in drug delivery, and how biomedical imaging tools can be used to evaluate tumor barriers and predict drug delivery to tumors and antitumor response.

## INTRODUCTION

The tumor microenvironment (TME) is characterized by many factors including increased concentrations of extracellular matrix proteins such as collagen, hyaluronan, and fibronectins, as well as low extracellular pH, tortuous neovasculature, and hypoxia^[1]^. This environment plays an essential role in tumor development and response to therapy, and is often highly heterogeneous even within the same tumor type and the same patient^[1]^. The gold standard of cancer treatment is chemotherapy; however, the effectiveness is limited due to heterogeneity of the TME itself, multidrug resistance, and nonspecific drug distribution^[2]^. The likelihood of a chemotherapeutic crossing tumor vasculature to act on cancer cells is inherently decreased due to physical barriers created based on these varying factors in the TME. To overcome these barriers, there has been an increasing trend toward utilizing nanotechnology. Nanotechnology has many theoretical advantages in regards to cancer treatment including prolonged exposure, increased solubility, targeted delivery, and overall improved therapeutic index^[3]^. There is also an expected increase in permeability when using nanotechnology due to their nanometer size and the association with the enhanced permeability and retention (EPR) effect. In theory, the EPR effect is caused by leaky neovasculature and defective lymphatic drainage^[4,5]^. However, the factors that affect EPR and the true presence of the EPR effects in preclinical tumor models and especially in patients with solid tumors are unclear. In addition, it is possible that the EPR effect is highly variable across tumor types and within patients.

A few nanoparticle agents have been developed and approved by the United States Food and Drug Administration (FDA) for the treatment of solid tumors including PEGylated liposomal doxorubicin (Doxil®), liposomal daunorubicin (DaunoXome®), liposomal irinotecan (Onivyde®), and paclitaxel albumin-bound particles (Abraxane®)^[6,7]^. Although treatment response has improved with the development of these nanoparticle drugs, in many cases the theoretical advantages of these drugs are not fully realized. The lower than expected efficacy of nanoparticles may be a result of the overall low tumor uptake of these agents that is due to the heterogeneity of the components that comprise the TME^[4,5]^. The varying factors of the TME that contribute to this heterogeneity include angiogenesis, stiff extracellular matrix, high interstitial fluid pressures, hypoxia, acidic pH, and the presence of immune cells (e.g., macrophages). A characteristic of tumor growth is angiogenesis where the rapid growing neovasculature are incredibly fragile, tortuous or containing abnormal twists and turns, irregularly shaped, dilated, and highly permeable with increased geometric resistance^[8]^. This abnormal vascular network results in heterogeneous hypoperfused or necrotic areas within tumor tissue^[9]^ with variability in blood distribution leading to variability in drug distribution^[4]^. After extravasation of the nanotherapy agent through the tumor vasculature, the stiff extracellular matrix caused by activated cancer associated fibroblasts and upregulation of extracellular matrix proteins exists as a barrier to the diffusional movement of the agent through the interstitial space^[10]^. High interstitial fluid pressures also prevent extravasation of the nanotherapy agents where they are expelled into systemic circulation from the tumor periphery into adjoining tissues^[11]^. Hypoxia further prevents nanotherapy from reaching various parts of the tumor by causing irregular vasculature and poorly perfused regions^[12]^. Immune cells, such as macrophages and monocytes, have been shown to take up nanoparticles in circulation, tumor, and tissues, and also alter the delivery of nanoparticles to tumors^[13,14]^.

Many combinational therapies have been developed to improve the efficacy of nanoparticles; however, there are still several identified and unidentified factors and barriers in the TME that inhibit nanoparticle delivery to tumors^[15]^. The most promising approach to improving drug delivery and efficacy in solid tumors is to modify or “normalize” the TME. This also demonstrates the need for methods to evaluate the components of the TME. These methods may also allow for greater efficacy of nanoparticle agents and potentially personalized treatment methods for patients or specific tumors and monitoring of treatment response^[16]^. Table 1 summarizes the factors within the TME, their effect on nanoparticle delivery to solid tumors, and the imaging methods that can be used to measure these TME factors. The response of the TME to selected treatment methods is also an important factor to consider in determining continuation of treatment; however, this topic is beyond the scope of this review. In this review we will summarize current imaging methods used to identify TME factors that could affect nano-drug delivery in an effort to provide insight on evaluating and modulating these factors as ways to improve nanoparticle drug delivery and efficacy.

## IMAGING THE TUMOR MICROENVIRONMENT

Many cancer therapies can be more or less effective depending on the components of the TME. For example, the primary mechanism of radiation therapy is the generation of reactive oxygen species where hypoxic tumors are radiation resistant. Hypoxia and other extracellular matrix proteins including collagen also act as a barrier to some chemotherapy, resulting in further disease progression^[29]^. To evaluate the mechanism of resistance of the TME to therapy, nanoparticles have been engineered to be stimuli-responsive when they interact with changes in physiochemical parameters such as changes in temperature, light, reduction/oxidation enzymes, or pH. These drugs include functional computed tomography (CT) contrast nanoagents that respond to the acidic pH in the tumor microenvironment^[30]^ and glutathione^[31]^, and fluorescent nanoprobes utilizing quantum dots that are “switched on” when binding to matrix metalloproteinase^[32]^. The EPR effect in the TME has also been evaluated where tumor accumulation of fermuxytol iron nanoparticles imaged with magnetic resonance imaging (MRI) is correlated to tumor delivery of nanoliposomal irinotecan^[33]^. Other nanoparticles used in imaging can be targeted with tumor specific antigens to allow for monitoring of cancer nanotherapy including tumor associated macrophage targeted fluorescent nanoparticles (ferumoxytol-VT680XL)^[34]^, antigen specific targeted nanoparticles radiolabeled with indium-111 and imaged with single photon emission computed tomography (SPECT)^[35]^, positron emission tomography (PET) imaged ^64^Cu-labeled HER-2 targeted PEGylated liposomal doxorubicin for HER-2 positive metastatic breast cancer^[36]^, and targeted ultrasound contrast agents (or targeted microbubbles) for vascular endothelial growth factor receptor-2 or a_v_b_3_ used to image tumor neovasculature and aid in assessment of tumor malignancy and treatment response^[37]^. This allows the nanoparticle agents to be used for targeted delivery and interaction with the TME, resulting in increased accumulation, facilitated drug release, and an increase in uniform distribution^[38]^. This type of active targeting depends on the specific interaction of the designed nanoparticle with the targeted component of the TME. Tracking of cells that interact with the TME is also useful in determining their use for antitumor therapy where mesenchymal stem cells can be tracked using organic semiconducting polymer nanoparticles imaged with photoacoustic imaging^[39]^ and superparamagnetic iron oxide nanoparticles with MRI^[40]^. Targeted delivery and tracking facilitate the need for a method to easily determine if the targeted or altered conditions are present in the TME and monitor the changes in these factors during and post treatment to determine the effectiveness.

In this section, promising imaging methods for characterization and monitoring of the TME will be reviewed. Current imaging methods that are available for potential use in the clinic include MRI, PET, SPECT, CT, and ultrasound. In addition, other optical imaging methods including photoacoustic imaging (PAI), intravital microscopy (IVM), bioluminescence, fluorescence, fluorescence molecular tomography (FMT), and optical coherence tomography (OCT) will be discussed. The goal is to present a high-level overview of current imaging methods used to characterize the TME relative to nanoparticle drug delivery.

### Magnetic resonance imaging

#### Introduction

MRI allows for a nondestructive quantitative investigation of various aspects of the TME with good tissue contrast, spatial information, and sensitivity. Detailed, whole body anatomical images are obtained by employing a strong magnetic field and radio frequency (RF) electromagnetic waves. The protons in the human body normally have a scattered orientation; however, when they enter the magnetic field, the protons align with this field. The applied RF pulse causes the protons to become deflected by 90° and store the energy of the RF pulse. After the RF pulse is suspended, the protons relax to their normal state and emit their stored energy where the time it takes for this phenomenon to occur differs depending on the type of tissue. Contrast agents can also be used to increase resolution and target specific cell types. The disadvantages of MRI as a technique for evaluating the TME include high costs and long acquisition times. MRI has the ability to reveal tumor treatment response of various sizes of nanoparticles by evaluating the EPR effect of a tumor. Karageorgis *et al*.^[41]^ measured the functional parameters of eight different orthotopic tumor models which were correlated with optical imaging results of accumulation of three sizes of fluorescent nanoparticles. They found that comparing permeability and blood volume fraction with fluorescent accumulation of the nanoparticles allowed for differentiation of “EPR positive” and “EPR negative” tumors^[41]^. Magnetic nanoparticles can also be used to model colocalization of therapeutic nanoparticles, yielding valuable insight into the kinetics of nanoparticle distribution in tumor-bearing mice^[42]^. Ramanathan *et al*.^[33]^ also demonstrated that the deposition characteristics of ferumoxytol iron nanoparticles in tumors may predict tumor response to nanoliposomal irinotecan in patients with solid tumors. Patients received ferumoxytol iron nanoparticles and the deposition characteristics and iron levels were quantified to demonstrate the EPR effect within tumor lesions. Treatment with nanoliposomal irinotecan followed and the tumor levels were measured at two separate biopsy locations. A positive trend was observed between ferumoxytol iron lesion values and irinotecan levels as well as tumor response^[33]^. These studies demonstrate the ability of MRI measurements of the distribution of iron nanoparticles to predict response to nanotherapy.

#### Extracellular matrix proteins and immune cells

The development of advanced MRI techniques such as dynamic contrast-enhanced-MRI (DCE-MRI), diffusion-weighted MRI (DW-MRI), blood oxygen level dependent (BOLD) MRI, tissue oxygen level dependent (TOLD) MRI, and MRI-chemical exchange saturation transfer (CEST) have further increased the utility of MRI as an imaging modality for the TME. The use of DW-MRI, and various contrast agents in DCE-MRI, have allowed for the specific imaging of extracellular matrix (ECM) proteins including collagen^[43,44]^ and hyaluronan^[45,46]^. These ECM proteins are recruited to the TME through activated fibroblast cells which are known to migrate toward the tumor. Tracking of fibroblasts has been achieved with MRI by pre-labeling fibroblasts *ex vivo* and then using magnetic resonance and optical probes to image the labeled fibroblasts post injection. The results showed recruitment of fibroblasts to the tumor and migration of the fibroblasts throughout the tumor^[47,48]^. Mesenchymal stem cells (MSCs), a fibroblast-like cell found surrounding blood vessels, have also been shown to migrate towards tumors. These cells have potential for repair of diseased or damaged tissue and are inherently tumor-homing and immunosuppressive, displaying many pro-tumor roles after arrival^[49]^. Tracking of MSCs labeled with superparamagnetic iron oxide nanoparticles in both malignant gliomas^[40]^ and pulmonary metastases^[50]^, and determining their use as vehicles for anti-tumor therapy^[51]^ have been performed using MRI, showing their tumor-homing response. Further use of iron nanoparticles as MRI contrast agents has resulted in imaging of tumor immune cells, specifically tumor-associated macrophages (TAMs). TAMs are also recruited to the TME, promote tumor progression^[52]^, and have been proven to be involved in phagocytosis and clearance of nanoparticle chemotherapeutics, and the tumor delivery of nanoparticles^[53,54]^. In addition, increased TAM presence in several types of human cancer have been associated with increased vascular density and worse clinical outcome. Thus, selective TAM imaging could guide treatment decisions and serve as a biomarker for long-term prognosis. Ultrasmall superparamagnetic iron-oxide nanoparticles (USPIO) are virus sized nanoparticles with an iron oxide core and a hydrophilic carboxydextran coating which, along with their size, allow them a much longer circulation half-life and help determine their pharmacology^[55]^. Iron exposure helps to regulate expression of iron transport related proteins, which are correlated to macrophage polarization states, suggesting that iron oxide nanoparticles would be preferentially phagocytosed by the multitude of TAMs in the TME^[56]^. In a study by Daldrup-Link *et al*.^[55]^ results showed that FDA approved USPIO are preferentially phagocytosed by TAMs versus neoplastic tumor cells; however, uptake of the USPIO nanoparticles by the neoplastic tumor cells could be a confounding variable when preferentially targeting macrophages. Preferential accumulation of nanoparticles in TAMs as measured by MRI has also been used to study metastatic lymph nodes in prostate cancer^[57–59]^.

#### Tumor vasculature

MMPs are known to be involved in tumor angiogenesis and their activity has been evaluated *in vivo* using novel proteinase-modulated MRI contrast agents^[60–63]^. This novel proteinase-modulated MRI contrast technology was used in the development of theranostic nanoparticles (TNPs) which enabled enzyme-specific drug activation at tumor sites and simultaneous *in vivo* MRI of drug delivery. The particles used ferumoxytol conjugated to an MMP-activatable peptide conjugate of azademthylcolchicine. The TNPs resulted in significant antitumor effects and tumor necrosis, demonstrating the potential of a nanotemplate that integrates tumor specificity, drug delivery, and *in vivo* imaging in a single moiety^[63]^. Other studies of MMP activity have utilized signal-amplifiable self-assembling (19) F MRI probes^[64]^ and an activatable cell-penetrating peptide covalently linked to cyclic-RGD, an integrin a_v_b_3_ binding domain which takes advantage of the interaction of MMP-2 with integrin a_v_b_3_^[65]^. These a_v_b_3_, along with a_v_b_5_, integrins have more recently been imaged with superparamagnetic iron oxide loaded (SPIO) cRGD PEGylated polyion complex vessels in order to image the neovasculature in glioblastoma^[66]^. Modified SPIO in magnetoliposomes have also been used for targeting a_v_b_3_ integrins to study tumor angiogenesis^[67]^. Specific investigation of tumor vasculature can be achieved with various advanced MRI methodologies. The first is DCE-MRI which uses direct markers of angiogenesis such as a_v_b_3_ integrins^[68]^. The second methodology is dynamic susceptibility contrast (DSC)-MRI, which is the most commonly used MRI method for cerebral perfusion^[69–71]^. For both DSC-MRI and DCE-MRI, the contrast agent extravasates from the vasculature of leaky blood vessels in the tumor and the vessel size and density, vascular permeability and perfusion, and extravascular space can be assessed^[72–75]^. DW-MRI is also used for evaluation of tissue perfusion; however, very complex quantitative analysis is required for this approach^[71,76]^. In order to acquire tissue blood flow and volume, arterial spin labeling BOLD-MRI is used. In BOLD-MRI methodologies, the visibility of the contrast is based on the concentration of deoxyhemoglobin, which is paramagnetic, and works best in poorly oxygenated tumors^[77]^. BOLD-MRI has demonstrated changes in tumor oxygenation following induction of angiogenesis^[78]^ and maps of functional vasculature^[79]^.

#### Tumor hypoxia

**Figure 1.**
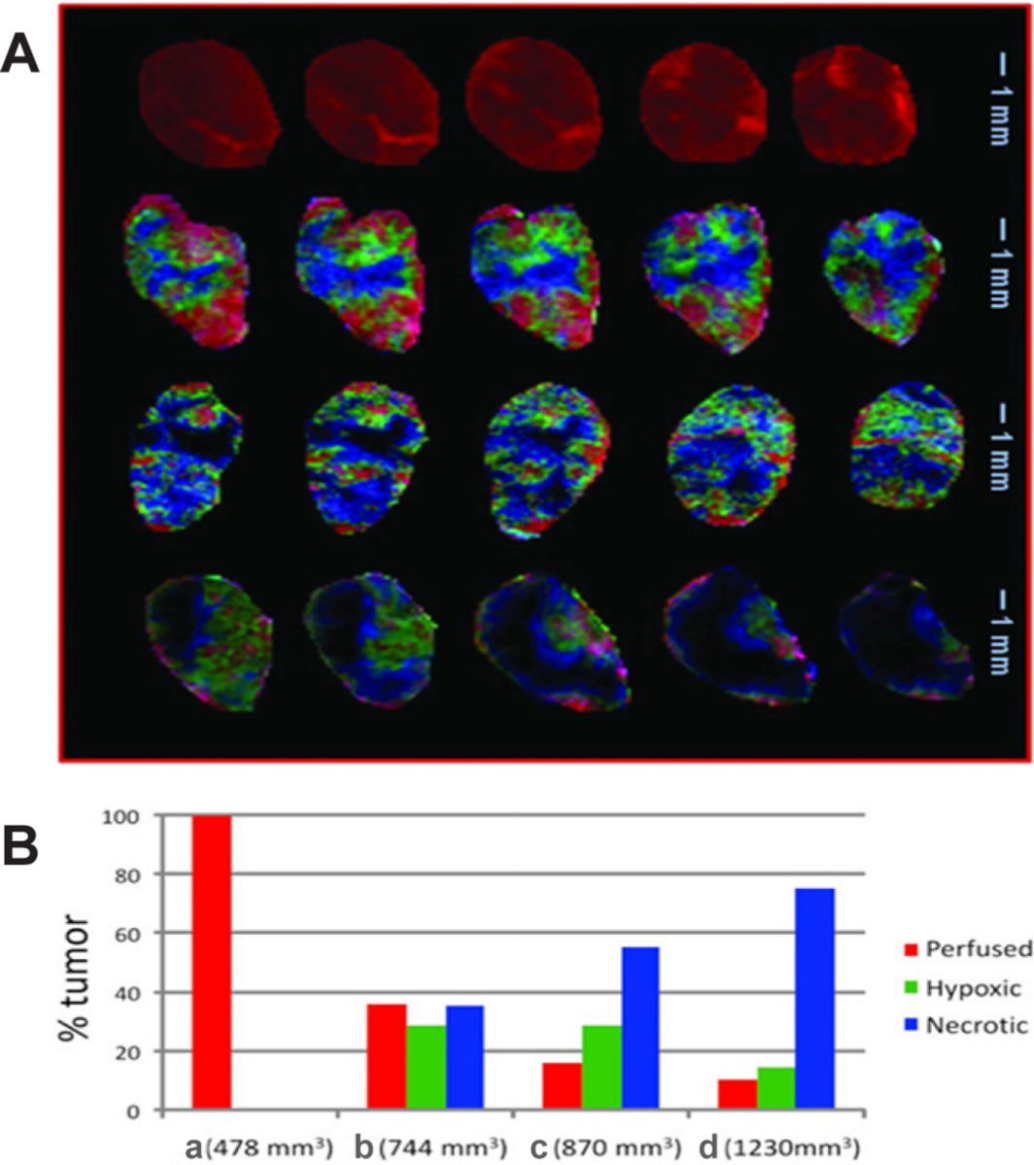
Study of well perfused, hypoxic, and necrotic regions of a prostate cancer tumor with dynamic contrast-enhanced mri with pattern recognition. (A) Composite color images of individual tumor subtissue features as identified by PR analysis. Images are presented for five tumor slices in four tumors with sizes: (a) 478 mm^3^, (b) 744 mm^3^, (c) 870 mm^3^, and (d) 1230 mm^3^. The perfused (red), hypoxic (green), and necrotic (blue/black) features are represented. (B) Tissue feature fractions in the four tumors. Figure reproduced with permission from^[85]^.

BOLD-MRI has also been used to image tumor hypoxia as a result of the leaky and tortuous vasculature. There are many clinical trials involving the use of agents to combat hypoxia and permit increased response to radiotherapy; however, tumor hypoxia for each patient should be measured in the clinic to determine eligibility for the studies. O’Connor *et al*.^[80]^ have used the fraction of tumor tissue refractory to oxygen challenge as a biomarker of hypoxia and demonstrated the ease at which this can be implemented in the clinic. A review of the comparison of BOLD-MRI and TOLD-MRI was completed by O’Connor *et al*.^[81]^ and described the theory and potential use of both methodologies to identify, spatially map, and quantify tumor hypoxia. Biomarkers derived from both BOLD-MRI and TOLD-MRI such as the dioxygen molecule (O_2_) and the deoxyhemoglobin monomer (Hb), can reveal underlying low pO_2_ and tissue hypoxia^[81]^. Several others have also demonstrated BOLD- and TOLD-MRI as prognostic biomarkers of response to therapy^[82–84]^. DCE-MRI has also proven useful for imaging tumor hypoxia where this methodology is routinely used for identification of tumors in patients, demonstrating the ease at which this could be implemented in the clinic^[85–87]^. Stoyanova *et al*.^[85]^ used an unsupervised pattern recognition technique which used a differential signal versus time curve associated with different TME characteristics in DCE-MRI data to differentiate between well perfused, hypoxic, and necrotic regions of prostate cancer. With DCE-MRI already routinely used for tumor identification, this technique could be easily translated to the clinic^[85]^ [Figure 1].

#### Glycolysis and tumor pH

Under the hypoxic conditions in the TME, glycolysis is the predominant pathway for energy production leading to decreased pH of the extracellular space and inducing resistance of chemotherapies that are weak bases^[88]^. This glycolysis has been profiled with MRI in pancreatic ductal adenocarcinoma by Yamamoto *et al*.^[89]^ and in lymphoma by Rodrigues *et al*.^[90]^ who used ^13^C-MRI. The resulting anaerobic conditions lead to decreased pH in the TME in which many MRI methods have been developed for imaging this gradient in pH. Gallagher *et al*.^[91]^ and Scholz *et al*.^[92]^ measured and quantified pH using ^13^C labeled bicarbonate. More commonly used for tumor pH imaging is CEST-MRI which selectively saturates exchangeable protons that are transferred to bulk water signal^[93,94]^. Chen *et al*.^[95]^ has demonstrated accurate tumor pH measurements with parametric maps of CEST-MRI data and Longo *et al*.^[96]^ demonstrated a CEST-MRI approach to measuring pH using an X-ray contrast agent. To remove the influence of concentration on pH measurements, paramagnetic CEST (PARACEST)-MRI can be used^[97–100]^. It is also possible to differentiate between intracellular pH and extracellular pH with CEST-MRI. Intracellular pH detection methods utilize amine and amide concentrations from endogenous tissue proteins, which predominately remain in the intracellular space^[101–106]^. Exogenous molecules have also been utilized as extracellular pH reporters, permitting monitoring the effectiveness of novel anticancer treatments which reverse the glycolytic tumor phenotype^[107–111]^. The ability to translate CEST-MRI to the clinic still requires significant optimization; however, preliminary results and continued optimization studies for reducing respiration artifacts and expand the coverage of the body during an acquisition show promising results^[96,112–117]^.

### Nuclear medicine

#### Introduction

The two most commonly used imaging modalities in nuclear medicine are SPECT^[118]^ and PET^[119]^. Both modalities allow visualization of metabolic processes by detecting gamma rays through use of radiopharmaceuticals. The radiopharmaceutical is conjugated to a biologically active molecule (typically a sugar used for cellular energy) and injected into the vasculature. The detectors capture emissions from the radiopharmaceuticals and use software to create tomographic images of the concentrations in the body. These radiopharmaceuticals in SPECT imaging emit gamma rays while the radiopharmaceutical in PET imaging emit positrons. SPECT imaging offers a significantly cheaper option in terms of equipment and radio tracer costs as well as the possibility of greatly increased imaging time due to SPECT tracers typically having a much longer half-life. In contrast, PET offers the advantage of higher spatial resolution images, and the ability to quantify metabolic activity for accurate assessment of therapeutic effect. Although PET offers more radio tracers available specific to molecules in the TME, their production also requires availability of a cyclotron^[118,119]^. PET also offers excellent sensitivity to small concentrations providing biochemical information of diseases including cancer through molecular imaging^[120]^. Detection of isotopes with PET can be obtained down to the 100 picomolar level in target tissues, where this low concentration has little or no physiological effect, allowing the independent study of the target mechanism^[121]^.

#### SPECT imaging

PET is more commonly used for cancer imaging and diagnosis; however, SPECT has been previously used to image MMPs^[62,122]^, MSCs^[51,123]^, hypoxia^[124]^, and immune cells^[125]^. SPECT has also been used to monitor cancer nanotherapies such as imaging of prostate specific membrane antigen targeted nanoparticles that were radiolabeled with indium-111 along with their untargeted nanoparticle. Results showed that the targeted nanotherapy revealed an accumulation of ~6% ID/g that remained constant over time whereas the untargeted nanoparticle had a higher tumor uptake of ~8% ID/g but was cleared more quickly between 48–96 h^[35]^. Other biodistribution studies of cancer nanotherapies demonstrate the increased circulation time of IV administered PEGylated liposomes^[126]^ along with the selective accumulation of technetium-99m (^99m^Tc)-radiolabeled Caelyx® in the tumor compared to surrounding tissue^[127,128]^.

#### PET imaging

Similar to SPECT, PET offers the ability to image track the biodistribution of cancer nanotherapy including the analysis of ^64^Cu-labeledHER2-targeted PEGylated liposomal doxorubicin in patients with HER2 positive metastatic breast cancer showing their long circulation time and decreased accumulation in healthy tissue. The tracking of this nanotherapy also showed large heterogeneity of tumor accumulation in primary breast carcinomas and in different metastatic lesions^[36]^. This tracking of nanotherapy is an important application of PET and further demonstrates the application of imaging and characterization of the TME in determining factors dictating the response to nanoparticle treatment. In other experiments, the application of PET zirconium-89 nanoreporter to predict response to nanotherapy was investigated. This study showed correlation of the tumor uptake of zirconium-89 nanoreporter with the tumor uptake of various other nanoparticle drugs. This demonstrated that the zirconium-89 nanoreporter could be used as a surrogate measure of nanoparticle tumor delivery and used as inclusion criterion for patient’s tumor that is amenable to nanotherapy^[129]^. PET has also been used for imaging MMPs^[130–134]^, MSCs^[135–138]^, and immune cells^[139–141]^. Choline-phospholipid metabolism PET imaging has been demonstrated by several groups and successfully translated to the clinic, despite limitations in metabolite discrimination^[142–145]^. PET imaging is also very useful for measuring tumor vasculature and lymphatics through quantitative measurement of blood flow and perfusion^[146–148]^, blood volume and vascular permeability^[149,150]^, and specific molecular markers of tumor vasculature including integrins^[151–156]^, VEGF receptors, and EGF receptors^[157–165]^.

#### Tumor hypoxia and pH

PET offers much more advanced imaging of tumor hypoxia^[166,167]^. The most commonly used PET tracer for hypoxia imaging is FMISO^[168]^ which has been used in patients with glioma^[169,170]^ and in mice with micrometastases^[135]^. When compared with other PET tracers for imaging tumor hypoxia, studies showed that each tracer had advantages that could be used depending on the desired imaging requirements^[171,172]^. PET imaging also has the capability of detecting changes in hypoxia before and after treatment, providing insight on treatment response^[173–175]^. Various materials have been investigated for use in PET hypoxia imaging including copper^[176]^ and gallium^[177]^, where copper demonstrated faster accumulation while gallium demonstrated minimal liver accumulation and excellent specificity to hypoxic tissue. Changes in hypoxia and glycolysis in the TME result in changes in pH where a few PET probes for pH have been developed; however, research in this area is limited due to the limited spatial resolution^[178–181]^. Under hypoxic conditions, glycolysis is the major energy producing pathway making glycolysis an attractive target for therapeutic interventions. Moreover, glycolytic inhibitors that cause ATP depletion show promising anticancer activity and regressions in *in vivo* models of solid tumors^[182]^. Monitoring glycolysis using PET has been demonstrated; however, only the early stages of glycolysis are probed, where more characterization is needed to evaluate complete metabolism^[89,183–185]^. Nuclear medicine has demonstrated broad use in imaging multiple factors within the TME, further demonstrating the abilities for correlating factors within the TME to successful delivery of nanotherapies^[129]^.

### Computed tomography

#### Introduction

CT is a noninvasive tomographic imaging technique based on X-ray attenuation that permits high efficiency and fast temporal resolution related to the electron density of tissue. The X-ray images are generated with a fan-shaped X-ray beam and digital X-ray detector that rotate about the patient to collect multiple X-ray projections needed to generate a tomographic CT image. The image processor then performs massive calculations to construct the CT image. A limitation of CT in imaging soft tissue lesions such as tumors, is a lack of contrast between the soft tissue and normal tissue, therefore contrast agents are necessary to achieve accurate tumor detection^[186]^. Clinically, iodinated small compounds are used as a CT contrast agent, but are usually limited by short circulation time, potential adverse effects, and lack of specificity^[187]^. However, Sahani *et al*.^[188]^ used CT contrast agents allowing the assessment of tumor vascularity and perfusion in rectal cancer where tumors showed higher blood flow and shorter mean transit time which reversed after treatment with chemotherapy and radiation therapy. Additionally, the study indicated that initial high blood flow and short mean transit time typically responded poorly to chemotherapy and radiation therapy^[188]^. To improve circulation time, specificity, and visualization of CT contrast agents in the tumor vasculature, nanoparticle contrast agents have been explored, allowing not only investigation of the tumor vasculature, but also how the nanoparticles interact with the TME^[189–191]^. Ghaghada *et al*.^[191]^ showed that the longer circulation time of nanoparticle CT contrast enabled longitudinal tracking, revealing regions with high ‘leakiness’ and increased vessel permeability within tumors. The extravascular signal enhancement of these nanoparticle CT contrast agents showed highly heterogeneous signals within all tumors and also varied between animals^[191]^. In recent years, there has been even more advancement in CT contrast agents with the development of negative CT contrast agents as well as spectral CT, making it feasible to use functional CT contrast nanoagents (FCTNAs) for evaluation of the TME^[192–194]^. These FCTNAs can be targeted to specifically bind to overexpressed receptors in the TME for imaging purposes and for targeted drug delivery. Zou *et al*.^[195]^ used targeted CT contrast to accumulate at an a_v_b_3_-integrin positive B16 melanoma, which once degraded, released the loaded doxorubicin resulting in suppression of the B16 melanoma. Targeted CT imaging has also been achieved for different cancer types, including human hepatocellular carcinoma through binding to the overexpression of asialoglycoprotein receptors^[31]^, 4T1 breast tumors through overexpressed p32 cell surface receptors^[196]^, and for the over- or under-expression of a_v_b_3_ integrin in various tumor types^[197]^.

#### Tumor hypoxia and pH

**Figure 2.**
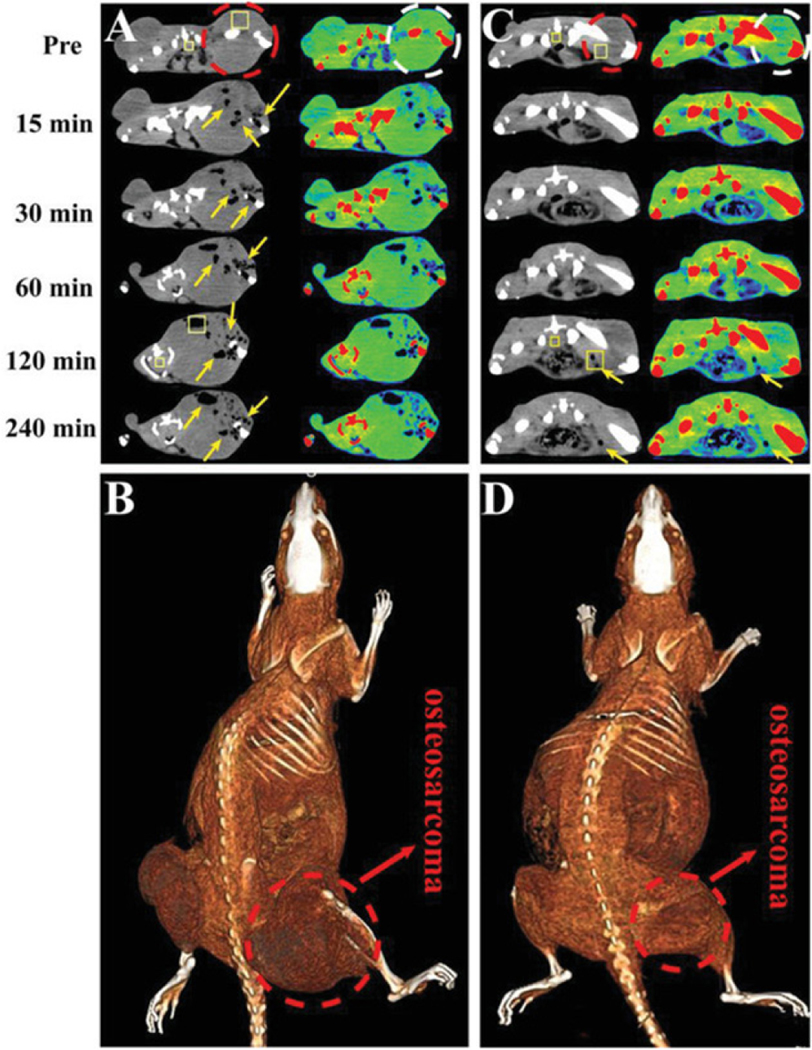
Computed tomography (CT) images using a negative CT contrast agent designed to generate hydrogen in response to acidic pH in the tumor microenvironment of osteosarcoma. (A, C) CT images of osteosarcoma-bearing rats by intratumor and intravenous injection of HMSN@AB@PEG nanoparticles (at various time points). Transverse gray and corresponding pseudo color images were obtained with a dashed line representing the area of osteosarcoma and the region of arrows was the low CT density area of H_2_ release. The rectangle ROI were used to calculate the variation of CT density between two time points (Pre and 120 min). (B, D) Corresponding intratumor and intravenous 3D reconstruction maps of rat with a dashed line representing the area of osteosarcoma. Figure reproduced with permission from^[193]^.

Another use of FCTNAs involves contrast that is designed to diagnose, determine different levels of, and treat tumor hypoxia, offering improved prognosis and decreased treatment resistance^[198–201]^. When a tumor is hypoxic, this often leads to changes in pH where FCTNAs have been developed to respond to the acidic microenvironment of the tumor. Tian *et al*.^[30]^ developed a FCTNA that, through interaction with the acidic TME, folds and inserts into the lipid bilayer of the tumor cells. Negative CT contrast agents have also been designed to generate hydrogen in response to the acidic TME for accurate diagnosis of osteosarcoma^[193]^ [Figure 2]. In order to enhance the effectiveness of pH responsive contrast agents, FCTNAs have also been conjugated to respond to glutathione^[31,202,203]^. Although conventional CT may be limited, it has great advantages of high spatial and temporal resolution and new nano-CT contrast agents have improved the CT technology allowing for improved monitoring of the TME, although it is important to note that relatively high local concentrations of contrast are needed to achieve good sensitivity^[204]^.

### Ultrasound

#### Introduction

Ultrasound is a widely used diagnostic tool for visualizing the structure of native tissues which uses backscattered radio-frequency sound waves to generate a gray-scale image, resulting in a qualitative image of tissue structure. The ultrasound beam is created through mechanical oscillations of crystals in a transducer that is excited by electrical pulses and converts the energy into sound. The sound waves are detected as reflected echoes after propagation through tissue where the reflections occur at the boundary between tissues with varying densities. This leads to the altered physical tissue characteristics in disease states being readily visualized. Conventional and advanced ultrasound techniques are excellent probes for monitoring and correlating factors within the TME due to cost effectiveness, ease of use, lack of ionizing radiation, non-invasiveness, portability/accessibility of the equipment, and the ability to multiplex. Recent work has focused on the use of nanotechnology in combination with ultrasound to increase the localized delivery of drugs to solid tumors^[205]^. The leaky and tortuous vasculature within the TME prevents accumulation of the nano-drug; however, an ultrasound process called sonoporation can increase the size of pores in the cell membrane after mechanical impact of ultrasound radiation, increasing permeability and allowing passive entry of nanoparticles into cells^[205]^. Ultrasound can also be used to release drug from nanoparticles. For example, ultrasound induced shear stress can be used to rupture nanoparticles (e.g., drug loaded polymersome, perfluorocarbon nanoemulsions, and liposomal nanocarriers), releasing their cargo at the targeted site^[206]^. Thus, sonoporation of the endothelial cells and shear stress of the nanoparticle drugs after interaction of ultrasound radiation allows for increased cell permeability, cell retention of the drug, stimulation of drug release only at the targeted site, and decreased off-target toxicity^[205,207]^. The field would benefit from increased depth imaging and improved quantification to allow increased translation of these advanced techniques into the clinic. Over the past several decades, many researchers have developed techniques to improve the ability to make tissue characterization with ultrasound quantitative^[208–211]^. Lizzi *et al*.^[212]^ and Ghoshal *et al*.^[208]^ performed analytical investigations of specific tissue features that determine spectral signatures. These advancements in ultrasound imaging have resulted in successful quantification of collagen, a major component of the TME and barrier to drug delivery to tumors^[213–216]^.

#### Tumor vasculature

**Figure 3.**
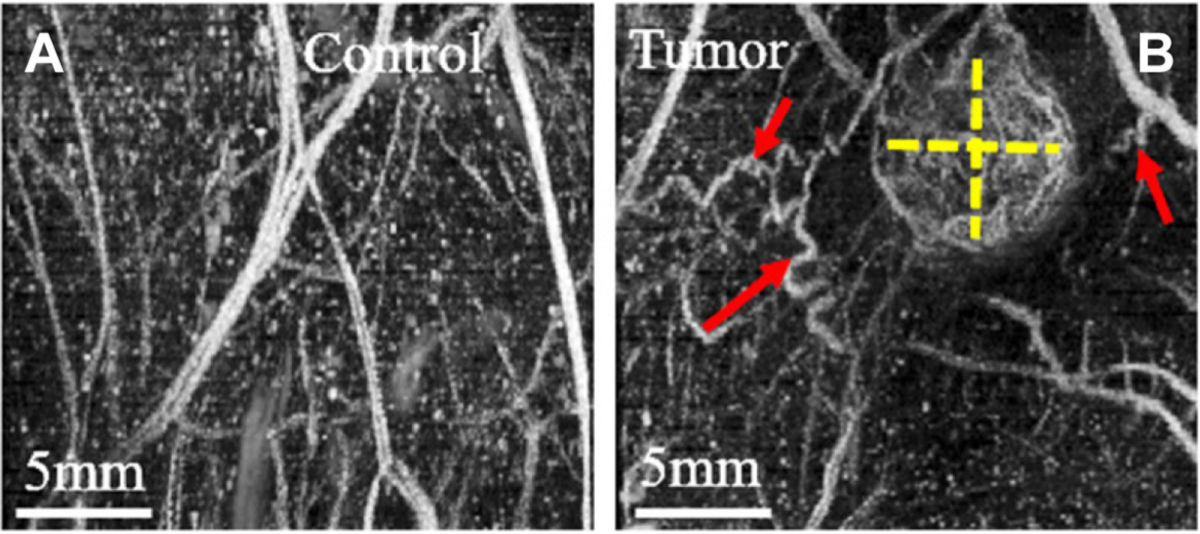
Acoustic angiography images of angiogenesis and vessel tortuosity. (A) Images of control non-tumor bearing regions and (B) fibrosarcoma tumor bearing regions in a rat. Tumor size is represented with dashed yellow lines. Tortuous vessels denoted by red arrows represent angiogenesis around the periphery of the tumor. Higher vascular density in the tumor is demonstrated by more enhancement in the images due to higher contrast agent perfusion. The vasculature in control images is more linear and directional, as shown. Figure reproduced with permission from^[217]^.

Other advancements in ultrasound imaging including the development of acoustic angiography based on the use of ultrasound contrast agents, called microbubbles, have resulted in visualization of tumor microvascular architecture without a significant contribution from background tissues^[218]^. Microbubbles are spherical cavities with a gas encapsulated in a shell made of phospholipids, surfactant, denatured human serum albumin, or synthetic polymer which resonate with ultrasound waves. Microbubbles provide a strongly reflective interface, and are currently clinically approved for use as an ultrasound contrast agent in cardiac and liver imaging^[219]^. These microbubble contrast agents are confined to the blood stream when injected intravenously. Acoustic angiography has been used to map micro-vascular networks in the TME^[218,220–222]^, differentiate tumor tissue from healthy tissue^[217,221]^ and various tumor types^[223]^, as well as monitor response to therapy^[37,224]^. The dense and tortuous vessel network in solid tumors allow the use of acoustic angiography in the assessment of neo-vascularization for early diagnosis^[217]^ [Figure 3]. One limitation of acoustic angiography for vascular imaging is the imaging depth (up to several centimeters^[218]^). However, this can be overcome by using techniques such as Doppler-based ultrasound techniques^[225]^ and ultrasound localization microscopy^[226]^, although these methods are susceptible to tissue motion. In order to increase specificity of ultrasound imaging to target molecules, microbubbles conjugated to antibodies or ligands are used where the molecular ultrasound targets are expressed on vascular endothelial cells. These microbubbles have greatly reduced adverse effects when compared to CT/MRI contrast agents with an added advantage of real-time imaging^[37]^.

MMPs are involved in angiogenesis, tumor invasion, and metastasis where ultrasound microbubbles have been conjugated with specific antibodies for MMP imaging^[227]^. Other angiogenic markers are vascular endothelial growth factor receptor 2 (VEGF-2) and a_v_b_3_ integrin receptor, which are both hallmarks of the neovasculature that occurs in tumors at an early stage to establish an independent oxygen and nutrient supply. Targeted microbubbles for VEGF-2 have been developed where imaging studies show expression of the receptor in several different tumor models^[228–231]^. Along with molecular targeting of vascular markers, microvasculature imaging is also an excellent tool in assessment of tumor malignancy and response to treatment such as predicting response to therapy before measurable changes in tumor volume^[37]^. Vessel tortuosity, or vessels that contain abnormal twists and turns leads to abnormal or variable drug distribution of nanoparticle drugs within the tumor. Lindsey *et al*.^[232]^ have assessed the combined microvascular and molecular imaging in a technique termed molecular acoustic angiography, suggesting that increased distance from VEGFR2- and selectin-targeting sites showed decreased vessel tortuosity. Lakshman *et al*.^[233]^ performed screening studies of the tumor vasculature in the TME of an orthotopic mouse model of human pancreatic cancer and demonstrated heterogeneous microvascular distribution with high perfusion at the periphery of the tumor and a poorly perfused core.

#### Ultrasound elastography

Ultrasound elastography is an advanced ultrasound method available for assessing factors within the TME. Elastography has the ability to measure tissue pressure and stiffness by visualizing the shear modulus of tissue, an intrinsic property of soft tissue defined as the ratio of shear stress to shear strain. For measurement of shear modulus, tissue motion is induced by using quasi-state, harmonic, or transient mechanical source. The mechanical movement or spatial variation of the tissue response is measured with an ultrasound transducer in which a simplified or continuum mechanical model is applied to obtain shear modulus values^[234]^. The pressure gradient at the tumor margin exerts mechanical stress which creates an increase in shear modulus^[235]^. Relative to the study of cancer, initially, ultrasound elastography has been primarily used for differentiating benign tumors from malignant^[236–239]^; however, more recently it has been used for the study of various factors within the TME. The correlation of cancer associated fibroblasts in breast cancer with markers of aSMA and CD34, has been investigated using ultrasound elastography finding that aSMA was positively associated with elastography scores and elevated in malignant lesions, while CD34 was negatively associated with elastography scores and is downregulated in malignancies^[240]^. Several other studies have shown increased collagen fibers with increased shear modulus^[241–243]^. In regards to drug delivery, elastography has been used to map the relationship between shear modulus and drug delivery within the pancreatic ductal adenocarcinoma TME. Results showed that drug delivery was directly influenced by shear modulus and that the factors within the TME including collagen modulate the shear modulus values^[244]^.

### Optical imaging techniques

#### Introduction

Current clinically available imaging techniques including PET, MRI, and CT typically have low spatial resolution, therefore, it is difficult to use these techniques for any real-time *in vivo* analysis of genetic, molecular, and cellular events^[245–247]^. Optical imaging methods are those that use luminescent enzymes such as bioluminescence or fluorescent proteins and dyes where these have been largely used for *in vitro* and *ex vivo* applications in cellular and molecular biology. These methods have the advantage of speed and versatility as well as the lack of special equipment, allowing the technique to be performed in any research lab or clinic^[248]^. Although these techniques are limited by depth of penetration (i.e., millimeter range), the ability to image the functional dynamics of the TME relative to time and space would give information in predicting and monitoring response to therapy.

#### Photoacoustic imaging

**Figure 4.**
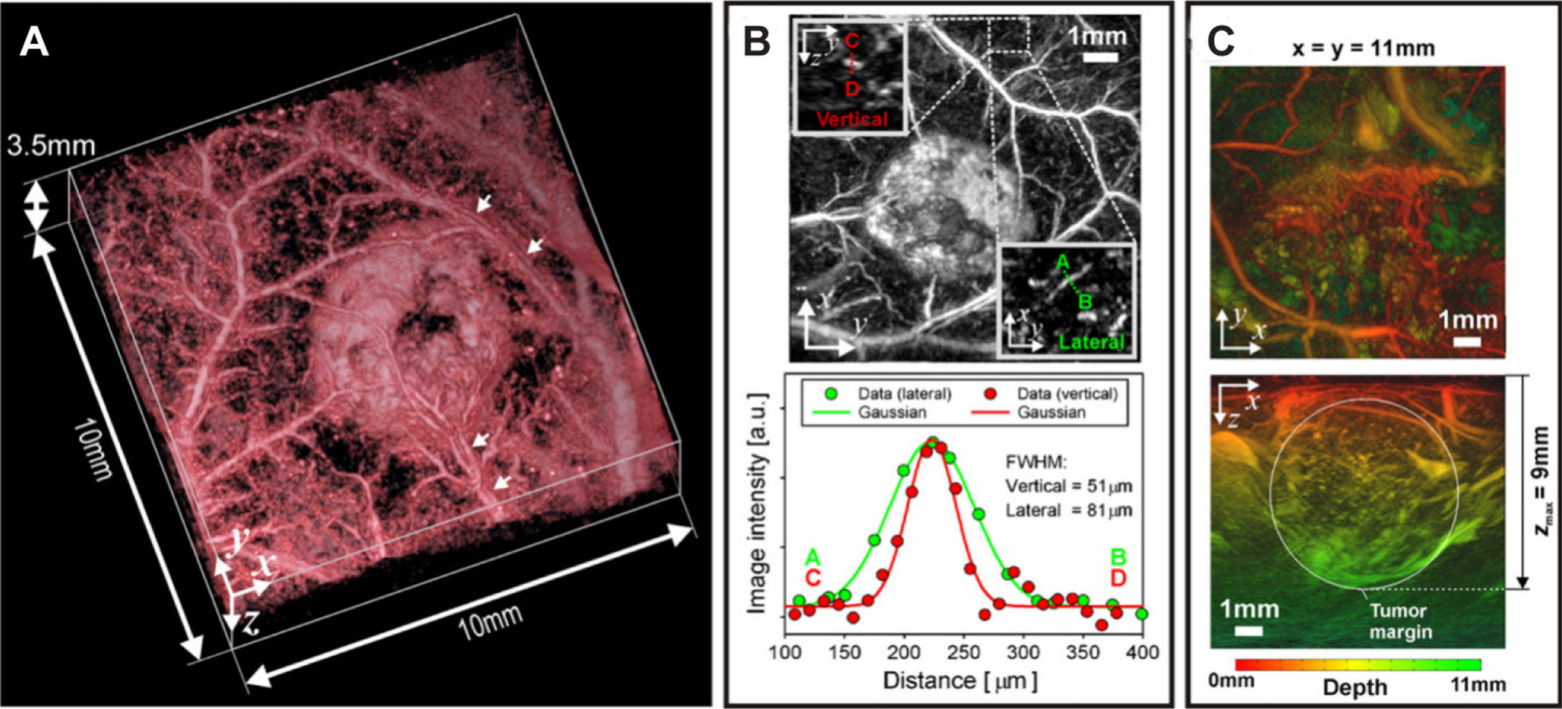
Tumor vasculature development and vessel tortuosity using photoacoustic imaging. (A) Volume-rendered 3-D photoacoustic image of a LS174T subcutaneous tumor (day 8 post inoculation) with arrows indicating artery-vein pairs. (B) Top: Maximum intensity projection (MIP) of the image presented in (A). The insets show magnified y-zy-z and x-yx-y MIPs of a region containing a small blood vessel. Bottom: Profiles across this vessel between points A-B and C-D were plotted and fitted with a Gaussian function to obtain spatial resolution. (C) Photoacoustic images of a larger SW1222 subcutaneous tumor (day 12 post-inoculation) illustrating an imaging depth of at least 9 mm. Figure reproduced with permission from^[250]^.

Photoacoustic imaging, or PAI, uses pulses of light to illuminate tissue, which causes a pressure change when absorbed, generating ultrasound waves that can be detected with an ultrasound transducer. To allow the spatial resolution, temporal resolution, imaging depth, and image contrast to be tuned in PAI, light sources varying in wavelength can be used. Wavelengths in the visible and near-infrared region primarily takes advantage of the contrast due to hemoglobin, although other contrast agents including dyes or genetically expressed absorbers are used to obtain targeted molecular contrast. PAI has been used to quantify nanodrug delivery by conjugating the particles with molecules that are oxidized during release to produce a concentration-dependent photoacoustic signal^[249]^. Additionally, PAI is used for visualizing the development of vasculature in tumors including tumor vessel tortuosity^[250]^ [Figure 4], vessel diameter and density^[251]^, and the recruitment of existing vessels to feed the tumor mass^[250,252]^. Tumor vasculature characteristics are imaged by using a single wavelength of light, selected as an isosbestic point of Hb and HbO_2_ at 532 nm and permits sub-100-mm resolution, non-invasively. Total hemoglobin concentration blood oxygen saturation (sO_2_) can be measured with PAI in combination with spectral unmixing algorithms where total hemoglobin concentration is typically higher and sO_2_ is typically lower in tumor tissue compared to normal tissue^[253–257]^. Low sO_2_ values are due to typical tumor hypoxia leading to poor perfusion and high consumption of O_2_ which can also be correlated with vascular maturity and perfusion efficiency^[258–261]^. In contrast, decreasing total hemoglobin concentration has been shown to indicate vessel normalization following anti-angiogenic therapies^[262]^ and increasing sO_2_ has been shown to predict radiotherapy response in mice^[263,264]^. Complementing the investigation of vascular features, tumor hypoxia^[265]^ and tumor pH^[95,266,267]^ can also be evaluated.

#### Extracellular matrix proteins and immune cells

Aside from tumor vasculature and oxygenation, many other components can be evaluated with PAI including collagen, MMPs, and immune cell infiltration. Preclinical studies of fibrosis and lipids has mainly focused on other diseases including Crohn’s disease^[268,269]^ and atherosclerosis^[270,271]^, but show excellent promise for its application in the TME. The monitoring of MMP production or activity in the TME provides a potential predictive biomarker of metastasis and their production has been monitored preclinically with PAI^[272–275]^. By using exogenous contrast agents, immune cells can be labeled and tracked, *in vivo*, and this has been shown with fluorescently labelled T cells^[276,277]^. Other contrast agents including organic semiconducting polymer nanoparticles and small-molecule dyes have been used to label and track injected stem cells^[39,278]^ where this pre-loading of cells avoids the macrophage phagocytosis of the nanoparticles^[279]^. The organic semiconducting nanoparticles in the far infrared window revealed significant photoacoustic contrast enhancement, demonstrating feasibility for real-time photoacoustic monitoring of stem cell behaviors such as cell differentiation which would advance the understanding of stem cell-based therapy^[39]^.

#### Intravital microscopy

**Figure 5.**
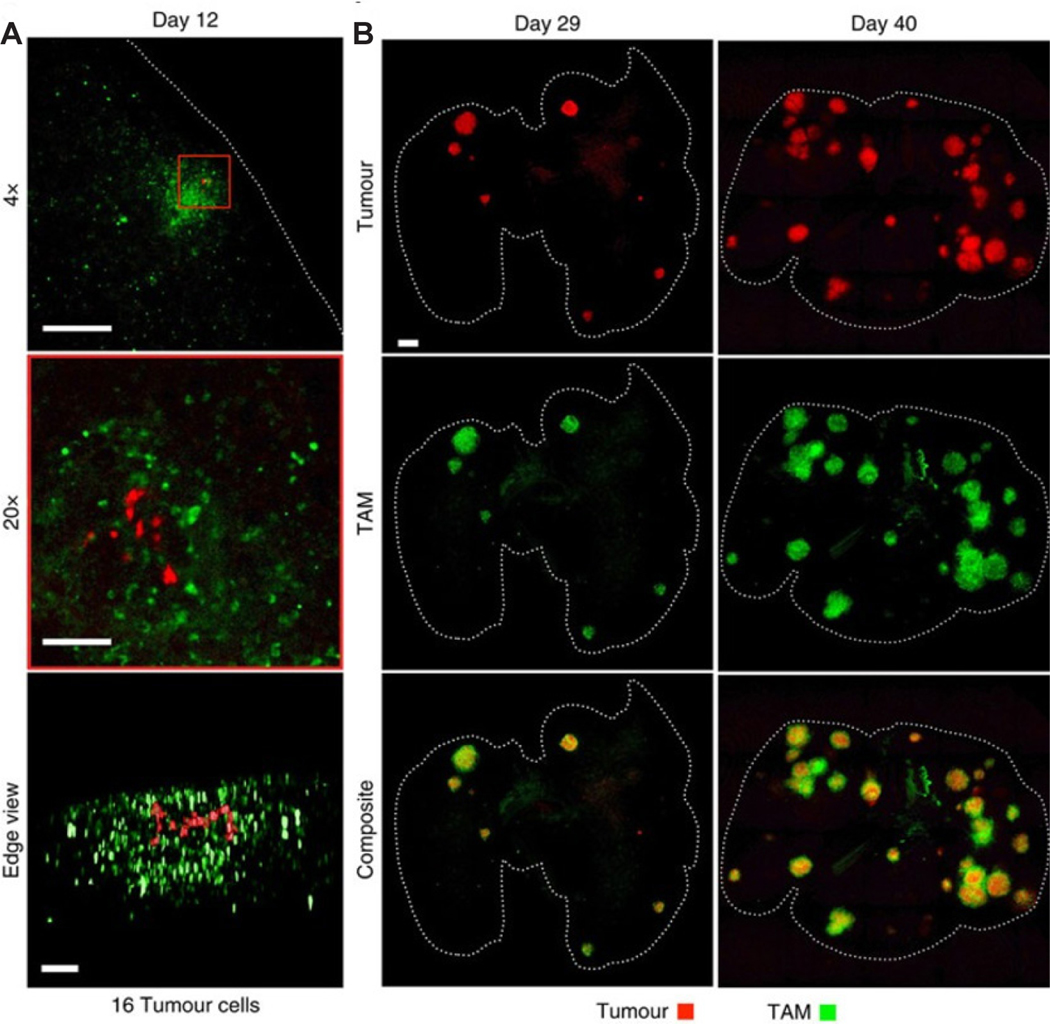
Investigation of the large heterogeneity in tumor macrophages and tumor size with intravital microscopy. (A). Early-stage tumors with clear indication of surrounding tumor associated macrophage (TAM) presence. TAM infiltration is observed in response to 16 individual tumor cells within a single nascent nodule. Scale bars, 1000 mm (top), 100 mm (middle/bottom). (B) Mouse tumor analysis at 2 stages of disease progression revealing facile tumor detection at varying locations and heterogeneous distribution of TAM. Dashed lines outline the lung. Scale bars, 1000 mm. For all, days denote time post inoculation. Figure reproduced with permission from^[34]^.

Intravital microscopy allows for *in vivo* imaging of cancer at subcellular-scale resolutions. This nonlinear microscopy relies on scanning tissue with single photon or multiphoton fluorescent microscopy and is commonly used to image the extracellular matrix in cancers^[280]^. To form the image, the excitation beam interacts with the target molecule to excite a transition between two energy levels. The light that is emitted upon relaxation of the target molecule is due to vibrational and rotational motion and this light is detected to generate the final image^[281]^. Most studies have been completed preclinically, with clinical applications being limited to endoscopic investigation of gastrointestinal cancers^[282]^ and cytoscopic investigation of bladder tumors^[283]^. Preclinically, advanced fluorescent labeling techniques can be used with IVM to characterize the TME in relation to tumor vasculature growth, regression, and density^[284,285]^, as well as metastasis^[286]^, tumor-associated immunocytes^[287]^, the interaction of cancer therapeutic agents with cancer and immune cells^[288]^, and the response of macrophages to neoadjuvant chemotherapy^[289]^. Often, a window of dorsal skinfold chamber is installed on animals with superficial tumors including skin tumors for IVM imaging, where deeper tumors such as colon, liver, or pancreatic tumors are not directly accessible, requiring specific surgical procedures to expose the tumor^[290,291]^. The heterogeneity of immune components such as macrophages can lead to large variances in prognosis. Cuccarese *et al*.^[34]^ studied the heterogeneous distribution of macrophages between tumors showing large heterogeneity between tumor sizes which was later correlated to nanotherapeutic drug delivery [Figure 5]. The authors found that the uptake of nanotherapeutics into the tumor were dependent upon macrophage density, where macrophage depletion decreased nanotherapy delivery and efficacy^[34]^. Macrophages have also been shown to mediate the antitumor activity of bisphosphonates in 4T1 mouse mammary tumors^[292]^. There have also been studies that employed nanotechnologies, referred to as “smart” nanotechnologies, that use fluorophores or quenchers which respond to changes in the TME conditions^[293]^. One limitation of IVM is overlap or bleed-through between related fluorophores and when using particular laser light sources and microscope filter sets while labeling and imaging more than one component of the TME. To overcome this limitation, fluorescence-lifetime imaging microscopy can be used in which fluorophores with different fluorescence lifetimes are used^[294]^ and this can be combined with standard wavelength filtering to allow investigation of TME dynamics^[295,296]^.

#### Bioluminescence imaging

Bioluminescence uses ultra-sensitive charge-coupled device cameras to measure biopermeable luminescence after administration of a luciferase gene such as _D_-luciferase of coelenterazine. Luciferases proteins are found naturally in insects, bacteria, and plants and have enzymatic activity that catalyzes the oxidation of the substrate in a reaction in the presence of ATP, oxygen, and luciferin resulting in the emission of a photon. The resulting photons can travel through tissue and thin bones and are detected to form the image. This process allows the visualization of the movement of cancer cells in living animals^[297–299]^. Bioluminescent nanoprobes have been developed for the *in vitro* analysis of MMP activity composed of AuNPs and luciferases, where the AuNPs efficiently quenched the bioluminescent emission allowing the nanoprobe to detect the proteolytic activity of MMP-2^[300]^. Other *in vitro* bioluminescent probes have been developed in combination with quantum dots allowing for multiplexing of different MMP activities and increased detection sensitivity^[301,302]^. *In vivo* bioluminescent detection of MMP activity has been achieved by using luciferase-quenched protein nanoprobes bound to collagen, allowing them to remain in the extracellular space for increased sensitivity^[303]^. Bioluminescent probes have also been used to track MSCs *in vivo*^[304–307]^, *in vitro*^[308]^, and *ex vivo*^[309]^, as well as immune cells^[310]^. Tumor hypoxia and altered pH were shown to reduce bioluminescent signals^[311,312]^ which led to the development of specific bioluminescent probes for tumor hypoxia using CYP450 reductase^[313]^ and a HIF-1a reporter construct^[314]^. Due to the advantage of bioluminescence for visualizing movement of cells, it is also an excellent tool for the analysis of tumor-stroma interactions and has been used with heterogeneous 3D models for drug screening^[315,316]^.

#### Fluorescence imaging

Fluorescence imaging excites the atoms in the target molecule to a higher energy level and when they relax to a lower level, they emit photons at a lower energy but longer wavelength than the excitation light. The emitted light is filtered from the excitation light based on wavelength before sent to the detector to form an image. Fluorescence imaging has an increased spatial and temporal resolution in comparison with bioluminescence imaging and has the ability to detect single cells in real time^[247,317]^. Fluorescent proteins can effectively transfect cancer cells, allowing visualization of the movement of cancer cells through vasculature, *in vivo,* demonstrating the ability to image processes such as metastasis and intravasation or extravasation of cancer cells^[318]^. By using multicolor fluorescent imaging, cancer cells can be distinguished from stromal cells in the TME^[319,320]^ and the interactions between the cancer cells and the tumor stroma can be elucidated^[321,322]^. Growth factors including the Ras superfamily G protein^[323]^ and VEGF which promotes angiogenesis^[324]^ have been fluorescently imaged, showing the promise of this method for analysis of cell signaling. Further studies of tumor angiogenesis using fluorescent imaging has been performed using a near infrared probe that labels blood vessels to evaluate changes over time^[325]^. Other fluorescent studies of tumor vasculature include combination with multispectral unmixing^[326]^, the use of Cy5.5-labeled probe for CD13 expression on tumor neovessels^[327]^, the use of a VEGF conjugated infrared dye^[328]^, and the use of dynamic fluorescent imaging for monitoring vascular density, perfusion rate, and permeability, simultaneously^[329]^. Similar to bioluminescence imaging, there are excellent fluorescent nanoprobes for imaging MMP activity that are “switched on” by the enzyme activity when they bind to the MMP target.

The design of these probes involves the use of quantum dots conjugated to peptides^[32,330,331]^ or the use of silica nanoparticles^[332]^ and gold nanoparticles^[331,333,334]^. Fluorescent probes have also been designed to investigate the accumulation of hyaluronan and collagen in *ex vivo* tumor sections^[335]^ and for *in vivo* intraoperative imaging of hyaluronic acid conjugates for assistance in surgical resection of pancreatic cancer^[336]^. Both MSCs and immune cells may be labeled *ex vivo* with fluorescent molecules to determine the localization and quantification of immune cells in whole tissue^[337]^ or to inject the prelabeled cells and view their activity *in vivo*^[338,339]^. Quante *et al*.^[340]^ showed evidence that CAFs originate from bone marrow and derive from MSCs through the expression of red fluorescent protein in aSMA-RFP transgenic mice. Often, investigations of CAFs are performed using immunofluorescence, *in vitro*^[341,342]^; however other studies have shown the expression of fibroblast activation protein on CAFs^[343]^. To study hypoxia in the TME, several fluorescent probes have been designed, primarily involving the use of near infrared probes conjugated to hypoxia activatable molecules, demonstrating a turn-on fluorescent probe^[344–347]^. Activatable fluorescent probes are also used to monitor pH in the tumor microenvironment^[348–351]^. Fang *et al*.^[349]^ designed a fluorescent probe that responds to the acidic nature of the tumor microenvironment by using a coumarin-hybridized dye with spirolactam ring structure which remains in a ring-closed form at neutral pH, displaying fluorescent peaks in the visible region but the rings opens up at acidic pH, displaying fluorescent peaks in the near infrared region. These probes are excellent for identifying the tumor. Liu *et al*.^[350]^ developed a fluorescent probe based on CdSe quantum dots where the reduced tumor pH leads to the loss of the surface stabilizer of the quantum dot, changing the fluorescence intensity and a mathematical model between fluorescence intensity and pH reveals the pH of the tumor environment within a range of 6.1–7.8. Optical imaging methods are advantageous in their increased spatial and temporal resolution as well as the ability to image multiple targets simultaneously; however, the depth of imaging is still limited as well as the topographical information.

#### Fluorescence molecular tomography

FMT takes advantage of the diffuse nature of photon propagation in tissue, producing a tomographic reconstruction in 3D through combining micro-CT and fluorescence images. Similar to fluorescence imaging, a fluorescent agent is injected and accumulated in targeted tissues. The fluorescent agent is excited with a laser, emitting photons with a longer wavelength than the excitation wavelength allowing them to be filtered and detected. With FMT, multiple spatial locations on the tissue are illuminated allowing a 3D reconstruction of the target tissue^[352]^. A main focus of FMT has been the imaging of MMPs including macrophages^[353]^. Activatable probes analyzed with FMT have been used to monitor the greatly upregulated MMP in glioblastoma by injecting a MMP-750 probe followed by acquiring a micro-CT and fluorescence images for the reconstruction of FMT^[354]^. The results allowed for accurate location detection of orthotopic glioma mouse tumors. MMPs have been shown to play a large role in angiogenesis, where FMT has been used to evaluate the volume of tumor vasculature in mice as well as the normalization of tumor vasculature over time after treatment with antiangiogenic agents^[355,356]^. A good correlation between the degree of tumor vascularization and the degree of tumor accumulation was found with FMT, suggesting that FMT can be used not only to characterize and predict enhanced accumulation of nanoparticle drugs but can be used to pre-select patients that are likely to respond to passively tumor-targeted nanomedicine treatments^[357]^. The advantages of FMT include its inherent quantitative nature, depth of penetration, ability to image without any ionizing radiation, and its ability to multiplex by using fluorophores that do not overlap all demonstrating the potential abilities of FMT for evaluation of the tumor microenvironment^[358]^.

#### Optical coherence tomography

OCT is a relatively new, non-invasive imaging technique that provides label-free imaging of living tissue. The contrast in OCT is due to the light scattering properties of cells, stroma, and other tissue structures and utilizes longer wavelengths in the near infrared region, which allows imaging at high resolutions deeper into tissues. The image is constructed by illuminating the sample and measuring the echo time delay and intensity of the backscattered light from the tissue. The tissue and boundaries causing the backscattered light have different optical properties and the dimensions of the tissue structure can be determined by measuring this backscattering time^[359]^. The mechanical components used for OCT can also be miniaturized, allowing them to be integrated into small probes, catheters, and endoscopes for imaging at internal sites^[291]^. In order to determine the longitudinal treatment response to nanotherapeutic delivery of rare ocular cancers, OCT was used and correlated with histopathology and flow cytometry, revealing relationships between neoplastic growth, neovascularization, and the immune microenvironment facilitating development of targeted therapies^[360]^. For investigating the TME, OCT has been used to investigate the collagen structures associated with bladder and skin cancer^[361,362]^ and to study the relationship between fibroblasts and mammary epithelial cells, *in vitro*, where the matrix stiffness was manipulated by changing the collagen content^[363]^. Assessment of the morphology of tumor vascular networks was performed using microstructural OCT^[291]^ and combining architectural and vascular OCT images acquired simultaneously allows for differentiating intra-tumor and peri-tumor vessels^[364]^. Other evaluations of vascular patterns have been performed in melanoma skin lesions with dynamic OCT^[365]^. The addition of contrast agents to OCT increases sensitivity, extending the capabilities into molecular imaging^[366]^ allowing for improved microvascular imaging^[367–369]^, investigation of the fundamental behaviors of tumor associated macrophages and other leukocytes through speckle-modulating OCT^[370]^ as well as the human epidermal growth factor receptor 2 (HER2 neu) protein with magnetomotive OCT which uses antibody conjugated magnetic nanoparticles^[371]^.

## CONCLUSION AND FUTURE WORK

Factors within the TME are typically investigated individually in preclinical studies and the imaging modality most appropriate for that specific or desired TME target is used. However, the TME is highly variable and there are dynamic interactions between the TME and cancer cells that affect tumor growth, development, and response to nanotherapies^[372]^. Thus, there is an urgent need for improved analysis of interactions and crosstalk within the TME, which requires advanced imaging techniques. As described above, standard imaging modalities currently used in the clinic are CT, MRI, PET, SPECT, and ultrasound where there are both advantages and disadvantages to these techniques when evaluating the tumor microenvironment. MRI, PET, and SPECT are very expensive to use and have long acquisition times; however, they provide excellent sensitivity and soft tissue contrast. PET and SPECT also require exposure to radiation. CT is not as costly and allows rapid, whole-body imaging with high resolution but has low soft tissue contrast and similarly requires exposure to radiation. Ultrasound is only semiquantitative with low soft tissue contrast but has the advantages of being a real-time imaging method with rapid, high spatial, and temporal resolution along with low cost. Other advancements in ultrasound imaging, including the use of contrast agents and the development of photoacoustic imaging, have demonstrated greatly improved soft tissue contrast. The continued development of safe and effective contrast agents will permit improvements in all clinical imaging modalities^[373]^. Optical imaging methods including PAI, OCT, FMT, intravital microscopy, fluorescence, and bioluminescence have a much higher sensitivity for contrast agents and a much broader availability of probes permitting detection of multiple TME components, but are limited by imaging depth, small field of view, and difficulty in quantitation^[374]^. There is an inherent tradeoff between imaging resolution and depth of penetration and the optimal selected method will be dependent upon the desired imaging goal and target. A summary of the imaging methods discussed with their advantages in resolution and imaging depth as well as safety concerns can be found in Table 2.

Currently, there is not one single imaging technology that can simply screen multiple TME factors in a single scan. Thus, to identify the dominant factor that alters tumor delivery of nanoparticles, multiple imaging technologies or varying contrast would need to be applied to each tumor in a similar fashion to immunohistochemical staining of tumor samples to evaluate a series of TME factors. In order to better select the appropriate imaging technique, it is important to know the advantages and disadvantages of the method based on the specific molecular target. To image extracellular matrix proteins MRI, OCT, and PAI have been used where OCT and PAI are limited by imaging depth, often leading them to be invasive and limiting their clinical translation; however, the development of MRI contrast to image various extracellular matrix proteins has demonstrated clinical feasibility although somewhat limited by resolution^[381]^. For visualizing immune cells and mesenchymal stem cells, MRI combined with iron oxide-based nanoparticles has lower sensitivity but permits repeated monitoring - a limitation in PET and SPECT - without pre-labeling with reporter genes as in fluorescence and bioluminescence imaging, although these methods lead to increased sensitivity^[382]^. Vascular function and distribution has been investigated with MRI, PET, CT, ultrasound, and optical imaging methods. The optical imaging methods (fluorescence, bioluminescence, intravital microscopy, OCT, and PAI) permit submillimeter resolution, visualizing microvasculature and blood flow but are invasive and limited by imaging depth. MRI, PET, and CT reveal whole body imaging but require the use of radioactive tracers, limiting serial monitoring and lack anatomical information, while ultrasound offers micron spatial resolution, the ability to reveal immature vasculature, and is low cost, it is not suitable for whole body imaging^[383]^. PET imaging is the preferred method for tumor hypoxia and pH imaging due to the wide availability of probes, although MRI offers lower cost (but decreased sensitivity) and optical imaging probes offer increased sensitivity but smaller imaging depth while being unavailable in the clinic^[384]^.

Nanoparticle pharmacology, and especially intra-organ or intra-tumor pharmacokinetics are becoming increasingly complex requiring higher quality imaging data which could be generated with multimodal imaging methods such as MRI with intravital microscopy^[33]^. The MRI plus intravital microscopy demonstrated that the uptake of ferumoxytol iron nanoparticles significantly correlated with reduction in tumor size when treated with nanoliposomal irinotecan^[33]^. Many studies focus on systemic nanoparticle pharmacokinetics or pharmacodynamics^[385]^ but fail to link an understanding of nanoparticle delivery with the response. Thus, there is a need for further studies of the interaction of nanoparticles with the TME in addition to simple correlation with tumor growth. Nanoparticles designed to respond to various factors within the TME such as immune cells^[386]^ or tumor vasculature^[387]^ will respond differently and should be studied separately or, more importantly, systemically in conjunction with multiple other factors in order to optimize nanoparticle treatment of solid tumors. There are also differences in the TME of different tumor types and primary lesions versus metastatic lesions, which should all be considered and evaluated when selecting nanoparticle agents for the treatment of patients with solid tumors.

## Figures and Tables

**Table 1.** Summary of tumor microenvironment factors, their effect on drug delivery to tumors, and characterization methods for each factor

TME factor	Effect on tumor drug delivery	Characterization method

Extracellular matrix proteins: collagen, proteoglycan (hyaluronan), fibronectin, and lamanin	Contribute to tissue density and stiffness, and creating a physical barrier to drug penetration^[17]^	OCT, PAI, and MRI*
Matrix metalloproteinase	Upregulates anti-apoptotic molecules protecting cancer cells from chemo-induced apoptosis^[18]^	Optical imaging (fluorescence, bioluminescence), PET, SPECT, MRI, PAI, and FMT*
Mesenchymal stromal (Stem) cells	Can differentiate into various cell types and protect cancer cells from external aggression allowing them to escape apoptosis^[19]^	Fluorescence, bioluminescence, MRI, PET, and SPECT
Cancer associated fibroblasts	Produce extracellular matrix proteins creating a physical barrier to drug delivery^[20,21]^	MRI
Immune cells	Down regulates proapoptotic molecules and upregulates interleukin-6, protecting tumor cells from chemo-induced cell death^[22]^	MRI, PAI, fluorescence, ultrasound, CT*, PET, SPECT, and bioluminescence
Tumor vasculature and lymphatics	Upregulates VEGF, modifying the apoptotic signaling pathway and expressing anti-apoptotic proteins^[23]^ and has varied and disorganized blood flow^[24]^ leading to variability in drug distribution	MRI, CT, PET, ultrasound, PAI, intravital microscopy, OCT, fluorescence, and bioluminescence
Metabolic-choline- phospholipid metabolism	Increases levels of glutathione, reducing ROH species resulting in chemoresistance^[25]^	MRI and PET
Hypoxia	Induces radio- and chemo-resistance through the mechanism of reactive oxygen radicals that damage DNA and down-regulate expession of DNA topoisomerase II^[26]^	PET, MRI, and CT
Glycolysis	Leads to increased production of lactic acid and H+ inducing hypoxia and reduced pH^[27]^	PET
pH	Influences drug uptake based on the acidity of the drug, protonating basic chemotherapeutics or allowing accumulation of acidic chemotherapeutics^[26]^	CT, MRI, PET, fluorescence, bioluminescence, and PAI
Tumor-stroma Interactions	Abnormal context of the TME facilitates abnormal cross-talk allowing tumor cells to disregard rules and adapt to the multicellular environment^[28]^	Fluorescence, intravital microscopy, MRI, and PET

* MRI: Magnetic resonance imaging; PET: positron emission tomography; SPECT: single photon emission computed tomography; CT: computed tomography; PAI: photoacoustic imaging; FMT: fluorescence molecular tomography; OCT: optical coherence tomography.

**Table 2.** Summary of imaging methods discussed including resolution, depth of imaging, contrast used & safety, quantitation accuracy, clinical translation, and the tumor microenvironment factors the imaging methods have been used to investigate.

Imaging method		Resolution	Imaging depth	Contrast agents & safety	TME factors	Accuracy in quantitation	Clinical translation

Magnetic resonance	Magnetic resonance imaging^[375,376]^	25–100 mm	Whole body	Gd- or iron-oxide-based probes and dendrimer-based macromolecules; conventional MRI is safe for imaging for patients without embedded metals in their body while GD-based contrast may cause some adverse health concerns	ECM proteins, matrix metalloproteinase, mesenchymal stromal cells, cancer associated fibroblasts, immune cells, tumor vasculature, metabolic-choline- phospholipid metabolism, hypoxia, pH, and tumor stroma interactions	Only semi-quantitative, relying on regional differences in signal intensities, and primarily used to reveal gross morphological abnormalities	Currently clinically used technique, making future TME studies feasible; however, some preclinical studies using higher strength magnetic fields may pose challenges
Nuclear	SPECT^[375,376]^	1–2 mm	Whole body	Radiolabeled antibodies, antibody fragments, and antigens; SPECT requires exposure to radiation	Matrix metalloproteinase, mesenchymal stromal cells, and immune cells	Although clinically only semiquantitative, quantitation is still a key benefit of nuclear medicine and can be improved with quantitative SPECT (only tested preclinically)	Currently a clinically available technique; however, clinical translation suffers from increased attenuation and decreased resolution
	PET^[375,376]^	1–2 mm	Whole body	Radiolabeled antibodies, antibody fragments, and nutrients, as well as activatable probes; PET requires exposure to radiation	Matrix metalloproteinase, mesenchymal stromal cells, immune cells, tumor vasculature, glycolysis, hypoxia, pH, and tumor stroma interactions	Similar to SPECT, quantitative accuracy can be improved with advanced algorithms, but these have only been applied in preclinical imaging	Currently clinically used for evaluation of therapy response, making clinical translation feasible
X-Rays	Computed tomography^[375,376]^	50–200 mm	Whole body	Water-soluble iodinated probes; requires exposure to radiation	Immune cells, tumor vasculature, hypoxia, and pH	Accurate quantitation is a benefit of CT	Clinical translation will be limited by approval of appropriate contrast agents
Ultrasound	Ultrasound^[289,375]^	50–500 mm	mm-cm	Endogenous, targeted microbubbles; no safety concerns with conventional ultrasound and microbubble contrast is FDA approved for cardiac^[377]^ and liver^[378]^ imaging	Immune cells and tumor vasculature	Generally qualitative but use of contrast and mathematical algorithms have improved ability for quantitation	Clinically available technique where microbubble contrast is FDA approved for other types of US imaging, making translation of preclinical techniques feasible
Optical	Photoacoustic imaging^[379]^	5–300 mm	0.7–40 mm	Fluorophores, nanoparticles, and quantum dots; no safety concerns, can be used repeatedly on tissue	ECM proteins, immune cells, tumor vasculature, and pH	Generally qualitative but several Size and cost of laser sources, studies have shown realization building a prototype, and of quantitative information clinical trials limit translation
	Intravital Microscopy^[376,380]^	100 nm -1 mm	100–300 mm	Endogenous; requires a surgically implanted window	Immune cells, tumor vasculature, and tumor stroma interactions	Quantitation is difficult; however, methods are being developed to improve ability for quantitation	Only feasible for introperative guidance
	Bioluminescence Imaging^[375,376]^	3–5 mm	1–2 cm	Reporter genes; requires use of lentiviral vectors, although toxicity is low	Matrix metalloproteinase, mesenchymal stromal cells, immune cells, tumor vasculature, and pH	Quantitation is difficult, preclinical methods are being developed for improvements	Only ideally used as a preclinical technique, human tumors do not express luciferases
	Fluorescence Imaging^[375,376]^	2–3 mm	< 1 cm	Fluorophores, fluorescent nanoparticles; fluorescent imaging in the near infrared is biologically safe and fluorescent particles show little to no toxicity	Matrix metalloproteinase, mesenchymal stromal cells, immune cells, tumor vasculature, pH, and tumor stroma interactions	Quantitation is difficult and is only relative to other regions in the tissue	Translation feasibility lies in fluorescence guided surgery, in which clinical trials have been completed
	Fluorescence molecular tomography^[352]^	<1mm	1–2 mm	Near infrared dyes, quantum dots, and reporter genes; imaging with near infrared light is biologically safe; however, quantum dot composite material is toxic in elemental forms	Matrix metalloproteinase	Improves quantitation over fluorescence imaging but is still challenging	Clinical translation limited by the development of scanners for smaller and more superficial structures
	Optical coherence tomography^[359]^	1–15 mm	2–3 mm	Endogenous; no biological safety concerns	ECM proteins, immune cells, and tumor vasculature	Typically, quantitation requires specialized proprietary software, but methods are being developed to overcome this	Currently clinically used for ophthalmic imaging, clinical translation for TME imaging depends on development of compact probes
